# Clinical and translational mode of single‐cell measurements: An artificial intelligent single‐cell

**DOI:** 10.1002/ctm2.1818

**Published:** 2024-09-22

**Authors:** Xiangdong Wang, Charles A. Powell, Qin Ma, Jia Fan

**Affiliations:** ^1^ Shanghai Institute of Clinical Bioinformatics Shanghai China; ^2^ Fudan University Center of Clinical Bioinformatics Shanghai China; ^3^ Zhongshan Hospital Fudan University Shanghai Medical College Shanghai China; ^4^ Division of Pulmonary Critical Care and Sleep Medicine Icahn School of Medicine at Mount Sinai New York New York USA; ^5^ Division of Computational Biology and Bioinformatics Department of Biomedical Informatics College of Medicine The Ohio State University New York New York USA; ^6^ Department of Liver Surgery and Transplantation and Key Laboratory of Carcinogenesis and Cancer Invasion Ministry of Education Liver Cancer Institute Zhongshan Hospital Fudan University Shanghai China; ^7^ Institutes of Biomedical Sciences State Key Laboratory of Genetic Engineering Fudan University Shanghai China

**Keywords:** artificial intelligence, gene sequencing, medicine, multi‐omics, single‐cell biology

## Abstract

With rapid development and mature of single‐cell measurements, single‐cell biology and pathology become an emerging discipline to understand the disease. However, it is important to address concerns raised by clinicians as to how to apply single‐cell measurements for clinical practice, translate the signals of single‐cell systems biology into determination of clinical phenotype, and predict patient response to therapies. The present Perspective proposes a new system coined as the clinical artificial intelligent single‐cell (caiSC) with the dynamic generator of clinical single‐cell informatics, artificial intelligent analyzers, molecular multimodal reference boxes, clinical inputs and outs, and AI‐based computerization. This system provides reliable and rapid information for impacting clinical diagnoses, monitoring, and prediction of the disease at the single‐cell level. The caiSC represents an important step and milestone to translate the single‐cell measurement into clinical application, assist clinicians’ decision‐making, and improve the quality of medical services. There is increasing evidence to support the possibility of the caiSC proposal, since the corresponding biotechnologies associated with caiSCs are rapidly developed. Therefore, we call the special attention and efforts from various scientists and clinicians on the caiSCs and believe that the appearance of the caiSCs can shed light on the future of clinical molecular medicine.

The number of clinical studies based on single‐cell sequencing and multi‐omics is increasing, with rapid improvements of single‐cell measurements and analyses for more than two decades, since the poly(A) polymerase chain reaction was designed for unbiased amplification of cDNA representing all polyadenylated RNA in a single cell.^1^ The chromosomal aberrations and mutations of DNA in a single cell were identified using comparative genomic hybridization measurement.^2^ Furthermore, both DNA and RNA were isolated from a single cell to uncover genomic and transcriptomic profiles on the same single cell, followed by the evaluation of the cellular function.^3^ The concepts of single‐cell biology, single‐cell systems biology or “whole single‐cell mode” are proposed to discover and develop biomarkers and therapeutic targets.^4^ Of human multiple organs/tissues, the single‐cell transcriptomic profiles of peripheral immune cells benefit clinicians in understanding molecular mechanisms of unexpected and uncontrolled major outbreaks of infectious diseases, like coronavirus disease 2019.^5^ On the basis of the growing evidence and understanding, a new discipline and initiative of clinical single‐cell biomedicine (cscBioMed) is being developed by integrating single‐cell biology, single‐cell systems biology, and single‐cell based medicine.^6^

The cscBioMed covers the understanding of molecular pathogenesis, diagnostics, therapeutics, and monitoring of responses to therapy at the single‐cell level, not only leading to clinical observations of the disease from the body into the cell, but also from the knowledge of multimodal single‐cell biology into clinical application and of cell‐cell interactions and signaling functions into diagnostic and therapeutic products. For example, peripheral non‐exhausted precursors of CD8+T cells were found to become exhausted CD8+T cells in primary and metastatic tumours or expanded Treg‐FOXP3 or activated effector memory T cells to migrate into the tissues.^7^ These observations were derived from characterization of molecular phenomes and correlations between immune single‐cells harvested from primary colorectal cancer or corresponding adjacent normal tissues, mesenteric lymph node, liver metastases or corresponding adjacent liver tissues, ascites, and peripheral blood. The peripheral immune cells reflect the dynamic status of the host's systemic health and can be an indicator of the tissue microenvironmental immunity. However, one of the major challenges in applying single‐cell measurements for clinical readouts is the need to standardize and optimize the comprehensive molecular profiles of single‐cell multi‐omics as well as analyze and deliver clinical reports precisely and time‐efficiently. The present Perspective illustrates a concept of clinical artificial intelligent single‐cell (caiSC) as a model or working station, to translate the single‐cell measurement of peripheral immune cells into routine measures in clinical biochemistry of haematology and provide a broader and deeper understanding and diagnosis of the disease nature, severity, durations, and responses to therapy.

The caiSC is here coined as a system integrated with clinical informatics, molecular multimodal trans‐omics, and digitalized and automatic computerization at the single‐cell level, with a clear and headlined emphasis on “Clinical”. It is important to outline the approach to develop training and validation data sets to establish, confirm, and refine the model, based upon new data and/or new methodologies. The caiSC consists of a dynamic generator of clinical single‐cell informatics, artificial intelligent single‐cell (aiSC), samples for clinical biochemicals and haematology, clinical outputs/outcomes from the system, automatic machine‐learning capacity, as well as reference boxes (Figure 1). The principle of caiSC design is based on the theory of clinical bioinformatics to integrate clinical phenomics with molecular multi‐omics.^8^, ^9^, ^10^ The most important objective of the caiSC function is to deliver the qualitative and quantitative values of molecular significance‐associated phenomes and cell subtype categories,The aiSC can be a platform for mono‐ or multi‐functional and omic profiling analyzers, fully dependent upon the number and types of reference boxes. and provide the decisive impact for disease‐associated and specialized indications, diagnoses and predictions. The definition of caiSC is continuously enriched, updated and improved with an increasing knowledge of single‐cell biology and with the rapid development of single‐cell measurement.

**FIGURE 1:**
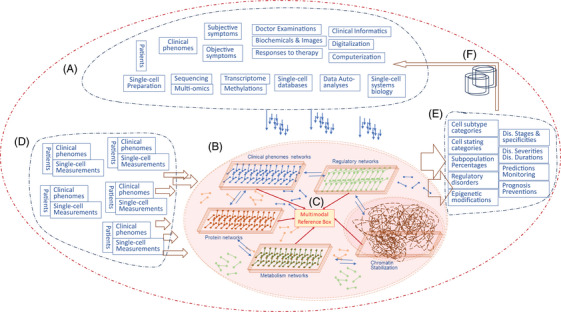
A schematic representation of the six critical sections of the clinical artificial intelligent single‐cell. (A) Dynamic generator of clinical single‐cell informatics as a source of clinical and molecular phenomes. (B) Artificial intelligent single cell with various reference boxes as an operator of comprehensive data analyses and integrations. (C) Intercommunication among multimodal reference boxes as a centre of multi/trans‐omic data crossing. (D) The input of clinical and molecular phenomes from the individual at the Department of Clinical Hematology & Biochemicals. (E) The outputs from the artificial intelligent single‐cell as the representatives of clinical outcomes. (F) The machine‐learning system with the capacity of re‐storing and remembering.

The general container serves as a constant resource of clinical phenomes, molecular multi‐omics, scoring systems and digitalized informatics from medical records and single‐cell measurements, after the standardization (Figure 1A). It contains raw data from established databases with auto‐learning capacities and simultaneously from new results of analysis (Figure 1F). Clinical descriptive phenomes are transferred into the digital and accountable information, which are highly dependent upon phenotype refinement, disease understanding and phenome specificity. The standardized procedures of clinical sample single‐cell measurements ensure the stable and repeatable generation of single‐cell multimodal data and the high quality and quantity of clinical and molecular phenomes before entering the aiSC (Figure 1B). A number of molecular multimodal omics data form multimodal reference boxes of individual molecules (Figure 1C), for example, clinical phenome networks, transcriptomic regulatory networks, protein interaction networks, metabolism networks, and chromatin stabilities, according to clinical and molecular characteristics and natures. After entering the aiSC, the clinical and molecular phenomes of each patient are screened, mapped, analyzed and identified, when passing through the corresponding reference box.

Differing from the concept of “whole cell model”, the aiSC has definite and specific reference boxes, which can be a box of transcriptomics, multiple boxes, or integrated boxes for rapid and precise analyses, a deep understanding of molecular interactions, and standardized productions. The aiSC handles the simultaneous analyses, inter‐box communications, and integrations of multiple modalities with various approaches (Figure 1C), for example, the multimodal reference boxes of the circulating immune system were constructed with the “weighted‐nearest neighbour” analysis.^11^ The number of reference boxes is increasing with the speed of method development, for example, single‐cell sequencing of RNA, DNA, ATAC, HiC, Methyl, T/BCR, ADT or CUT&RUN, while the quality is dependent upon the precision and automation of reference box analyses. The aiSC can be a platform for mono‐ or multi‐functional and omic profiling analyzers, fully dependent upon the number and types of reference boxes. Different from the concept of single‐cell biology, the aiSC contains a disease‐specific reference box to correlate the molecular and clinical phenomes and provide the molecular and subtyping characteristics of the disease's nature, severity, and response to therapy. Of those boxes, the reference‐mapping algorithms may present the special power and impact for defining the disease‐associated cell subtype populations, managing great variations of patient sources, and crossing molecular multimodalities.^12^ The strength and capacity of single or multiple reference boxes are highly dependent upon the volume of integrated platforms/databases of single‐cell measurements, the capacity of machine learning and deep mining, and the understanding of heterogeneity among cells.

The clinical application of the established caiSC provides new insights and approaches for clinical haematology and biochemicals, especially for the routine examination of peripheral blood immune cells. For example, the clinical phenomes and medical informatics of patients with lung diseases were digitalized by the digital evaluation scoring system based on the severity of each element and integrated with molecular phenomes of the corresponding individual sample.^13^ By this principle, the clinical phenomes, biological function and morphology, and transcriptomic profiles of the peripheral immune single cells can be harvested and input to the caiSC dynamically after routine measurements, as the new analyses of caiSCs (Figure 1D). Through analyses and integration of multimodal reference boxes, the categories of cell subtypes/subsets and functional states and percentages of subpopulations are reported within the time frame needed clinically (Figure 1E). The accuracy of each single‐cell subtype/state is highly dependent upon the recognition of the cell identity marker gene panels (ciMGPs) for representing the cell subtypes/states. The source of ciMGPs should be traced to define the molecular evidence of the nomenclature, the repeatability and overlaps of ciMGPs among subtypes/states be clarified to avoid false cell labelling, and the name and nomenclature of ciMGPs be standardized and recognized globally to unify the clinical report.^14^, ^15^, ^16^ The disorders of molecular regulations and epigenetic modifications are reported to indicate potential signal pathways‐associated pathogenesis and appearances of clinical phenomes (Figure 1E), by integrating and crossing the multimodal references boxes. One of the most important expectations from the caiSC is to deliver the information on disease nature, specificity, stage, severity, duration, prediction, and prognosis and assist clinicians’ decision‐making by linking and integrating with clinical phenomic reference boxes on diagnostic. Clinical phenotypes accompanied by reports of caiSCs will increase the accuracy and applications of traditional clinical practices with the goal of improving the quality of clinical practices and patient outcomes.

The caiSC is feasible with the capacity of artificial intelligence (AI), automatic machine‐learning and efficient algorithms and is able to ingest and generate comprehensive multi‐/trans‐omic profiles from the individual analysis of new data (Figure 1F). The generator can continuously enrich and empower the capacities of the general container (Figure 1A), by making the volume of data larger and the value of references more accurate. One of the great advances in caiSCs is the AI‐based computational algorithms through each part of caiSCs from the establishment of the dynamic generator which is the source of aiSC multimodal reference boxes and the receptor of new multimodal data from the patients consistently. The AI algorithms and strategies as the central motor can fuel the caiSC capacities of data collections, analyses, integrations, and reports more efficiently and precisely. On the basis of the principle of clinical AI modelling,^17^ the transparency and application of AI in caiSCs are refreshed by the clear and smart designs of six sections/modules in the caiSC (Figure 1). This design facilitates the separation and optimization of multimodal data for model training and testing of reference boxes, the evaluation and examination of caiSC performance and modelling, as well as the reproducibility and accuracy of outputs from caiSCs.

In conclusion, we propose a new system coined as the caiSC with the dynamic generator of clinical single‐cell informatics, caiSC, molecular multimodal reference boxes, clinical inputs and outs and AI‐based computerization. This system aims to provide reliable and rapid information for impacting clinical diagnoses, monitoring, and prediction of the disease at the single‐cell level. The caiSC represents an important step and milestone to translate the single‐cell measurement into clinical application, benefit clinicians’ decision‐making, and improve the quality of medical services. There is increasing evidence to support the possibility of the caiSC proposal since the corresponding biotechnologies associated with caiSCs are rapidly developed. Therefore, we call special attention and efforts from various scientists and clinicians on the caiSCs and believe that the appearance of the caiSCs can shed light on the future of clinical molecular medicine.

## AUTHOR CONTRIBUTIONS

Xiangdong Wang, Charles A. Powell, Qin Ma, and Jia Fan contributed equally.

## FUNDING INFORMATION

Not applicable.

## ETHICS STATEMENT

Not applicable.
